# In Vitro Hatching of *Scylla paramamosain* Embryos: Insights from Developmental and Transcriptomic Analyses

**DOI:** 10.3390/ijms27020714

**Published:** 2026-01-10

**Authors:** Zhiqiang Liu, Qi Gou, Xueyang Wang, Wei Wang, Lingbo Ma, Keyi Ma

**Affiliations:** 1 Key Laboratory of East China Sea Fishery Resources Exploitation, Ministry of Agriculture and Rural Affairs, East China Sea Fisheries Research Institute, Chinese Academy of Fishery Sciences, No. 300 Jungong Road, Yangpu Area, Shanghai 200090, China; 2 Ninghai Fishery Innovation Research Center, Ningbo 315604, China; 3 College of Fisheries and Life Sciences, Dalian Ocean University, Dalian 116023, China

**Keywords:** *Scylla paramamosain*, embryo, in vitro hatching, transcriptomics

## Abstract

*Scylla paramamosain* is a commercially important crab species widely cultured in China. However, artificial breeding remains limited by the high mortality of ovigerous females and asynchronous embryo hatching. In vitro embryo hatching has emerged as a promising alternative, yet its practical feasibility and underlying molecular mechanisms have not been systematically investigated. In this study, we examined the developmental characteristics of *S. paramamosain* embryos under different temperature regimes and hatching modes, evaluated embryo viability following maternal death, and compared transcriptomic profiles of Zoea I larvae between in vitro and maternal hatching. Our results demonstrated that temperature had a pronounced effect on embryogenesis and survival, with 27–30 °C identified as the optimal range for development and hatching. Both low and high temperature extremes markedly reduced embryo survival. Developmental trajectories were largely comparable between in vitro and maternal hatching, confirming the reliability and feasibility of the in vitro approach. Embryos collected within 4 h after maternal death exhibited high hatching success, whereas those obtained after 8 h failed to hatch. Transcriptomic analysis revealed 3505 differentially expressed genes, including 1933 upregulated and 1572 downregulated, which were significantly enriched in pathways related to cell cycle regulation, energy metabolism, immune defense, and ion transport. These findings implied that in vitro embryos could maintain developmental competence by stabilizing genomic integrity, reallocating energy resources, and activating stress responsive mechanisms. This study provides the first comprehensive evidence supporting the feasibility of in vitro embryo hatching in *S. paramamosain* and offers practical insights for optimizing temperature regimes, improving the utilization of maternal resources, and advancing large scale seedstock production in crab aquaculture.

## 1. Introduction

In vitro hatching refers to the cultivation of embryos outside their natural developmental environment, including the maternal body and egg capsule, under artificially controlled conditions of temperature, humidity, nutrient supply, and gas exchange. This method provides a stable and controllable platform for the direct observation of embryonic developmental dynamics. By mimicking maternal cues such as thermal and hormonal gradients, in vitro hatching enables detailed investigations of cell differentiation, organogenesis, and spatiotemporal gene expression, as exemplified by studies on the regulatory roles of *Hox* genes in axial patterning [1,2,3,4]. Consequently, in vitro hatching has become an important experimental approach for elucidating developmental mechanisms and evolutionary conservation in model organisms such as zebrafish and mice [1]. The integration of in vitro hatching with CRISPR-based gene editing provides a powerful framework for precise genetic manipulation and functional validation of embryonic genes through targeted knockouts and phenotype analyses. This combined approach not only accelerates the establishment of disease models but also facilitates research on directed stem cell differentiation and epigenetic regulation [3,4]. Furthermore, reliable assays for evaluating CRISPR editing efficiency, such as the cleavage assay, have been developed to rapidly and accurately assess gene-editing outcomes at the embryonic stage [2].

From a genetic breeding perspective, in vitro hatching serves as an important tool for improving both livestock and crop species through its integration with in vitro fertilization, embryo screening for sex determination and disease resistance, and interspecies chimerism. These applications markedly enhance breeding efficiency and shorten generational intervals, thereby accelerating the selection of desirable traits such as high yield and stress tolerance. In addition, in vitro hatching technologies are increasingly recognized for their potential contributions to species conservation. For instance, more than 65 ovum pick-up and in vitro fertilization procedures have been successfully performed in the northern white rhinoceros, resulting in the production and cryopreservation of viable embryos [5,6].

Currently, in vitro hatching techniques for fish species, such as zebrafish, Atlantic salmon, and tilapia, are relatively well established [7,8,9,10]. By combining in vitro fertilization with precise control of environmental parameters, including temperature, salinity, and dissolved oxygen, large-scale fry production can be achieved, thereby improving both the precision and efficiency of seedstock generation. In zebrafish, a well-developed in vitro embryo culture system has been successfully applied to identify key regulators of myogenesis [10]. Building upon this foundation, in vitro fertilization coupled with environmentally controlled hatching has been standardized for tilapia and Atlantic salmon. In tilapia, ovulation induced by GnRH-a and hCG, the use of chilled milt for fertilization, and hatching under thermostatically regulated and water-quality-controlled conditions consistently produce high-quality embryos and healthy fry, providing a reliable platform for large-scale seedstock production and mechanistic studies [8]. A recent study employing a single-dose hCG treatment further improved fertilization and hatching rates as well as early fry survival, facilitating the integration of in vitro hatching with trait screening and functional experimentation [9]. In Atlantic salmon, standardized chorion processing and protein profiling at defined accumulated thermal unit stages have enabled the use of chorion composition as a biomarker for embryo quality within in vitro hatching quality-control frameworks [7]. In contrast, research on in vitro hatching of economically important crustaceans such as shrimps and crabs remains limited, even though their embryonic development occurs externally. In vitro hatching studies are important for crustacean aquaculture and developmental biology because they enable precise control of key environmental variables (e.g., temperature and salinity), reduce broodstock-related losses (e.g., mortality of ovigerous females), and provide synchronized embryos for direct observation and stage-resolved molecular sampling. Recent studies in other decapods have demonstrated the utility of controlled hatching systems, for example, temperature-dependent embryonic development and hatching performance assessments in the swimming crab *Portunus trituberculatus*, controlled embryo development of the giant freshwater prawn *Macrobrachium rosenbergii* under different salinities [11,12]. However, a key unresolved question is whether maternal organisms provide additional energy resources or developmental regulation to embryos, which constrains the optimization of in vitro hatching conditions for crustacean embryogenesis. Consequently, most attempts at in vitro hatching in crustaceans have resulted in low survival or complete developmental failure.

*Scylla paramamosain* is one of the larger and faster-growing members of the family Portunidae. Owing to its delicate flavor and high nutritional value, *S. paramamosain* has become an important aquaculture species in the coastal regions of China [13,14]. However, compared with the Chinese mitten crab (*Eriocheir sinensis*) and the swimming crab (*P. trituberculatus*), for which artificial breeding and germplasm enhancement technologies have achieved substantial progress, artificial seedling rearing for *S. paramamosain* remains relatively underdeveloped. For *E. sinensis*, seed production techniques encompassing broodstock maturation, spawning, and larval rearing have supported the continuous supply of larvae and juveniles to aquaculture operations in China since the 1980s [15]. Similarly, for *P. trituberculatus*, studies on broodstock cultivation and offspring release have demonstrated that pond-reared broodstock perform comparably to wild populations in terms of reproductive performance and larval quality [16], and artificial seedling rearing has now reached an industrial scale [17]. In contrast, the seedstock supply of *S. paramamosain* remains heavily dependent on wild-caught larvae [18]. In China, the aquaculture and fishing yield of *S. paramamosain* exceeded 220,000 tons in 2022, indicating a large and growing demand for seedstock [19]. The availability and seasonal timing of these wild seed resources are strongly influenced by environmental factors such as temperature and rainfall, which constrain the sustainable and large-scale development of *S. paramamosain* aquaculture. Furthermore, several fundamental challenges continue to limit hatchery reliability. High larval mortality, often caused by pathogen outbreaks and fluctuations in water quality, remains a major bottleneck [20]. Consistent with this bottleneck, recent hatchery trials have reported that larval survival can be <2% under practical rearing conditions, underscoring the instability of large-scale seed production [21]. In addition, larval nutrition and feeding strategies are not yet standardized. Production still relies heavily on live feeds, while stage-specific requirements for essential fatty acids, including n-3 highly unsaturated fatty acids (HUFA) and the DHA-to-EPA ratio [22].

Overcoming the technical bottlenecks in artificial seedling rearing and achieving stable, large-scale seedstock output are critical for the sustainable expansion of *S. paramamosain* aquaculture. The seed production process typically involves broodstock conditioning, maintenance of ovigerous females and larval rearing. However, once females enter the berried stage, they often exhibit reduced mobility, decreased feeding activity, deteriorating physiological condition, and increased susceptibility to pathogenic infection. Consequently, the mortality of ovigerous females during embryogenesis is a common cause of failure in artificial seedling rearing [23]. Establishing normal embryonic development under in vitro conditions is critically important for supporting consistent, large-scale seedstock production by enabling the utilization of embryos from weakened or dead ovigerous females. Moreover, the successful establishment of in vitro hatching for *S. paramamosain* would provide a powerful platform for genetic improvement through selective breeding and rapid gene editing, thereby accelerating both applied breeding programs and fundamental research on developmental and functional genomics in this commercially important species.

In this study, we investigated the in vitro hatching of *S. paramamosain* embryos. Developmental progression was examined across successive stages, including cleavage, blastula, gastrula, non-segmented larva, compound-eye pigmentation, and membrane-enclosed zoea, under different temperature regimes. Survival to the zoea stage was assessed across temperature treatments, and transcriptomic analyses compared in vitro-hatched and maternally hatched embryos. These findings provide critical insights for developing efficient, large-scale seed production systems and establish a theoretical framework for the sustainable advancement of mud crab aquaculture.

## 2. Results

### 2.1. Developmental Timing of Fertilized Eggs at Different Temperatures

**Figure 1:**
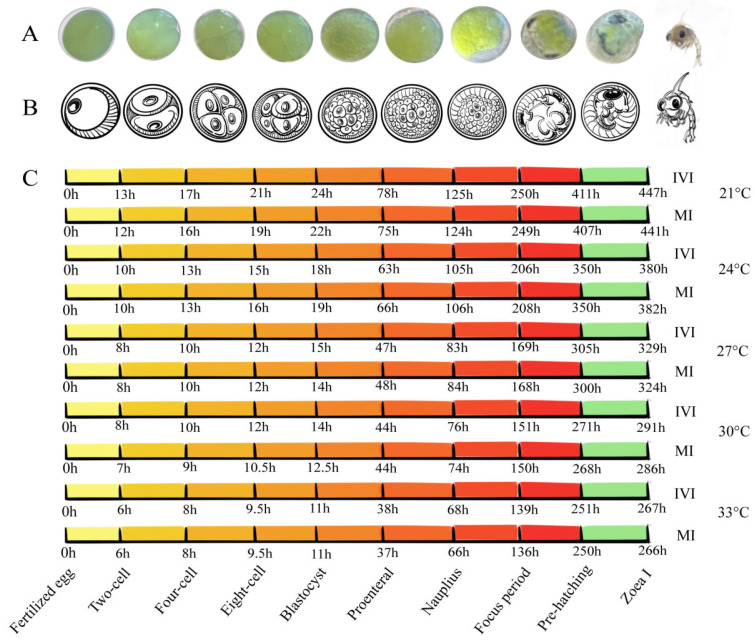
Development cycle of *S. paramamosain* embryo from fertilized egg to Zoea I under different temperatures. IVI: in vitro hatching group; MI: maternal hatching group. (**A**): Embryos from fertilized egg to Zoea I observed under a microscope. (**B**): Schematic diagram of embryos from fertilized egg to Zoea I. (**C**): Development time of embryos at different temperatures in vitro and in vivo.

The embryonic development of *S. paramamosain* progresses through a series of sequential stages, including the fertilized-egg stage, cleavage stage (2-cell, 4-cell, 8-cell, and subsequent divisions), blastula stage, gastrula stage, nauplius-like larval stage, compound-eye pigmentation stage, and intra-membranous zoea stage (Figure 1A,B) [19]. In the present study, no significant differences (*p* > 0.05) were observed in the time required to reach the corresponding developmental stages between maternal and in vitro hatching at the same temperature (Figure 1C), indicating that in vitro hatching does not affect the developmental rate of *S. paramamosain* embryos. However, developmental duration varied markedly across different temperatures. Embryonic development was slowest at 21 °C, requiring a total of 447 h to reach ZI. Development time progressively decreased at 24 °C, 27 °C, and 30 °C, whereas embryos incubated at 33 °C developed most rapidly, reaching ZI in 267 h. These results indicate that embryonic development in *S. paramamosain* is strongly temperature-dependent, with higher temperatures accelerating developmental progression.

The accumulated thermal units required for embryos to reach the ZI were compared between in vitro and maternal hatching conditions (Table 1). Overall, accumulated thermal units for the IVI group were slightly higher than those for the MI group (*p* > 0.05). At 21 °C, 27 °C, 30 °C, and 33 °C, IVI embryos required marginally higher accumulated temperature values than MI embryos. In contrast, at 24 °C, the MI group reached 9168 °C·h, exceeding the IVI value of 9120 °C·h by 48 °C·h.

### 2.2. Survival Rates of In Vitro-Hatched Embryos

**Figure 2:**
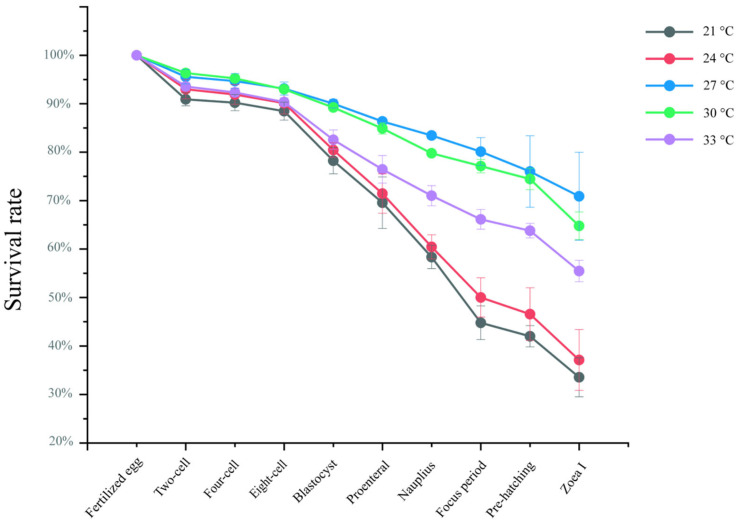
Survival rate of *S. paramamosain* embryo from fertilized eggs to Zoea I in vitro at different temperatures.

The survival rates of *S. paramamosain* embryos cultured in vitro at five temperatures (21 °C, 24 °C, 27 °C, 30 °C, and 33 °C) were evaluated across successive developmental stages. As shown in Figure 2, embryos at the 2-cell, 4-cell, and 8-cell stages exhibited high survival rates under all temperature treatments. However, after the blastula stage, survival declined markedly at 21 °C, 24 °C, and 33 °C (compared to 27 °C, *p* < 0.05), with the most pronounced reduction observed at 21 °C (compared to 27 °C, *p* < 0.05), where the survival rate decreased to 33% by the membrane-enclosed zoea stage (pre-hatching). Embryos hatched at 27 °C and 30 °C maintained relatively high survival (compared to 21 °C, *p* < 0.05), with the highest rate of 70% recorded at 27 °C at Zoea I (compared to 21 °C, *p* < 0.05).

**Figure 3:**
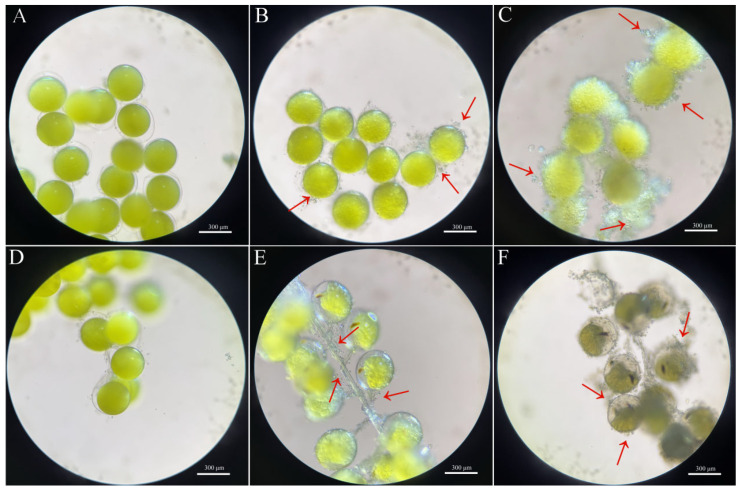
In vitro culture of *S. paramamosain* embryos at 18 °C (**A**–**C**) and 36 °C (**D**–**F**). Red arrows indicated protozoan colonies.

At 18 °C, embryonic development was slower than at 21 °C, with fertilized eggs requiring approximately 16 h to reach the 2-cell stage. Development arrested at the blastula stage, accompanied by the appearance and progressive proliferation of protozoan colonies on the embryonic membrane and setae, ultimately leading to embryo death. At 36 °C, embryonic development proceeded faster than at 33 °C, with fertilized eggs initiating cleavage within 4 h and rapidly reaching the compound-eye pigmentation stage. Nevertheless, extensive protozoan colonization on embryos and abdominal appendage setae resulted in heavy coverage and subsequent mortality (Figure 3). These findings indicate that both excessively low and high temperatures impair successful hatching of *S. paramamosain* embryos.

### 2.3. In Vitro-Hatched Embryos at Different Developmental Stages After Maternal Death

**Figure 4:**
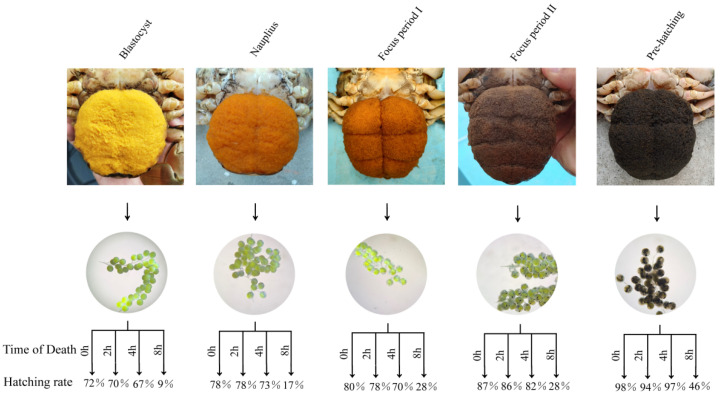
In vitro culture of embryos derived from the dead female *S. paramamosain*. Embryos were collected at 0, 2, 4, and 8 h post-mortem, with a sample size of 300 embryos per time point.

Embryos sampled from ovigerous females immediately after death (0 h) exhibited in vitro hatching rates comparable to those of embryos collected from live females and hatched in vitro. Compared with the 0 h group, embryos obtained at 2 h and 4 h after maternal death showed slight declines in survival and hatching success (*p* > 0.05), whereas those collected at 8 h exhibited a pronounced reduction (*p* < 0.05) (Figure 4). These results demonstrate that viable embryos could be harvested for a defined period post-mortem and successfully hatched in vitro to the ZI.

### 2.4. Transcriptome Sequencing and DEGs Analysis

**Figure 5:**
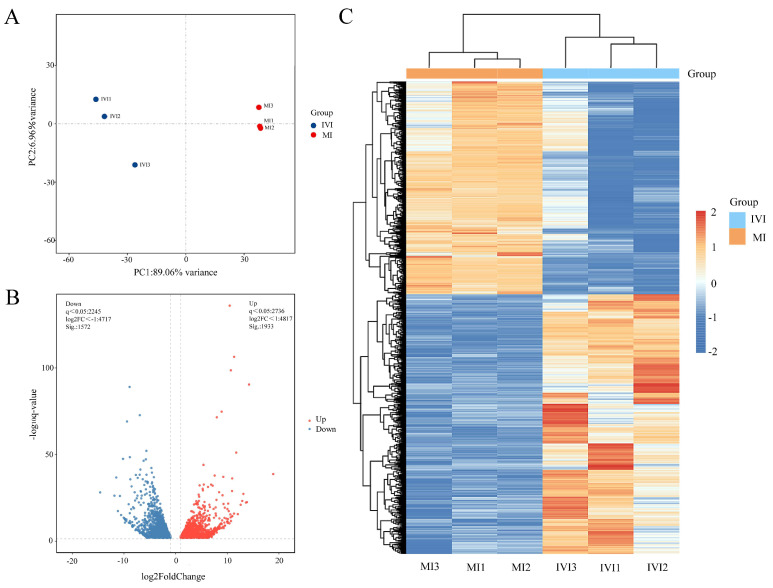
Integrated analysis of transcriptome data from in vitro and maternal hatching groups. (**A**) PCA score plots of transcriptomic datasets. The x-axis was the first principal component (PC1) and the y-axis was the second principal component (PC2). (**B**) volcano plot of differentially expressed genes (DEGs). Genes with significant differential expression were shown as red dots (upregulated) and blue dots (downregulated). (**C**) hierarchical clustering heatmap of DEGs. The x-axis showed sample names and their hierarchical clustering, and the y-axis showed hierarchical clustering of DEGs. Each cell represented one gene, and its color indicated expression level (red, high expression; and blue, low expression). IVI: in vitro hatching group; MI: maternal hatching group.

A total of 39.62 Gb of clean data were generated, with per-sample yields ranging from 5.84 to 7.06 Gb, Q30 base proportions between 96.03% and 96.74%, and GC contents exceeding 38.89% (Table 2). Principal component analysis (PCA) revealed high similarity among samples within each group (PC1 = 89.06%; PC2 = 6.96%), with variance primarily occurring between groups, indicating the reliability and consistency of the sequencing data (Figure 5A). Following data processing, 17,261 genes were identified. Differential expression analysis between IVI and MI groups revealed 3505 DEGs, including 1933 upregulated and 1572 downregulated genes in in vitro-hatched larvae compared with maternally hatched larvae (Table S1, Figure 5B). The heat map further illustrated distinct expression profiles between the two groups (Figure 5C).

### 2.5. GO Annotation of DEGs

**Figure 6:**
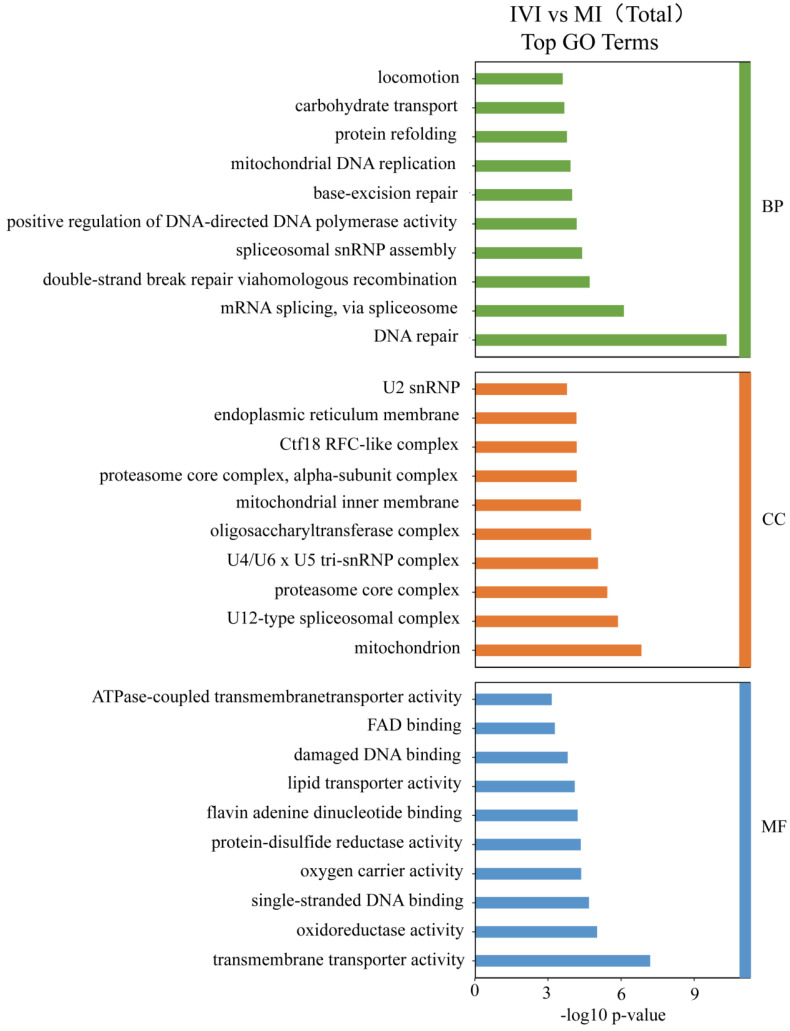
Top annotated GO terms from the comparison of in vitro hatching group vs. maternal hatching group. BP, Biological Process; CC, Cellular Component; MF, Molecular Function.

As shown in Figure 6, DEGs were significantly enriched in terms associated with DNA repair, mRNA splicing via the spliceosome, double-strand break repair via homologous recombination, base-excision repair, mitochondrial DNA replication, protein refolding, and locomotion in the biological process (BP) category. Among these, DNA repair-related terms exhibited the highest level of significance, indicating pronounced differences between the IVI and MI groups in mechanisms involved in maintaining genomic stability and responding to DNA damage.

Within the cellular component (CC) category, significantly enriched terms included the U2 small nuclear ribonucleoprotein complex, Ctf18 RFC-like complex, proteasome core complex, mitochondrial inner membrane, endoplasmic reticulum membrane, and oligosaccharyltransferase complex. Mitochondria and mitochondria-associated structures were prominently represented, suggesting differential expression and functional status of mitochondrial components between the IVI and MI larvae.

In the molecular function (MF) category, enriched GO terms included ATPase-coupled transmembrane transporter activity, FAD binding, damaged DNA binding, lipid transporter activity, oxygen carrier activity, and single-stranded DNA binding. The strong enrichment of transmembrane transport functions, particularly ATP-dependent transport, indicates potential differences between the two groups in material exchange and regulation of cellular homeostasis.

### 2.6. KEGG Pathway Enrichment Analysis of DEGs

**Figure 7:**
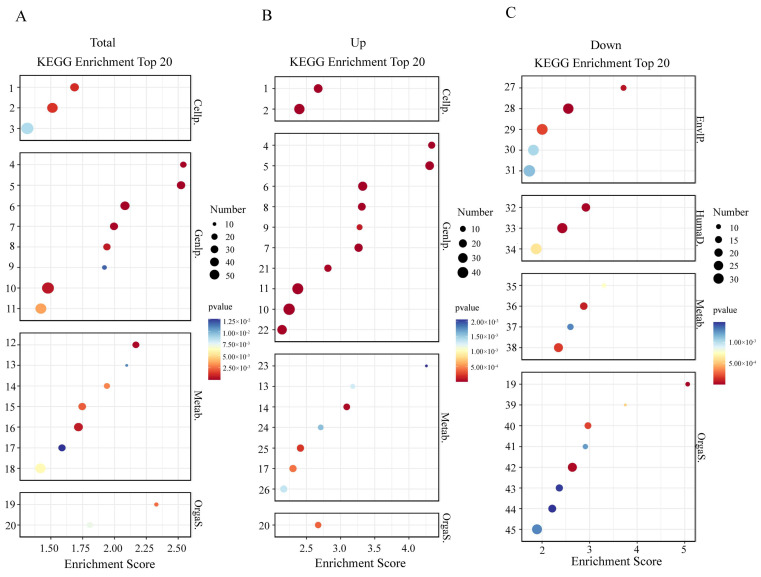
Total—(**A**), up—(**B**) and down—(**C**) top 20 KEGG pathway enrichment of differentially expressed genes (DEGs) from the comparison of in vitro hatching group vs. maternal hatching group. 1. Cell cycle-yeast (ko04111), 2. Cell cycle (ko04110), 3. Lysosome (ko04142), 4. Mismatch repair (ko03040), 5. DNA replication (ko03030), 6. Nucleotide excision repair (ko03420), 7. Base excision repair (ko03410), 8. Proteasome (ko03040), 9. Protein export (ko03060), 10. Protein processing in endoplasmic reticulum (ko04141), 11. Spliceosome (ko03040), 12. Alanine, aspartate and glutamate metabolism (ko00250), 13. One carbon pool by folate (ko00670), 14. Isoquinoline alkaloid biosynthesis (ko00950), 15. Glycine, serine and threonine metabolism (ko00260), 16. Amino sugar and nucleotide sugar metabolism (ko00520), 17. Tyrosine metabolism (ko00350), 18. Purine metabolism (ko00230), 19. Circadian rhythm (ko04710), 20. Antigen processing and presentation (ko04612), 21. Homologous recombination (ko03440), 22. Nucleocytoplasmic transport (ko03013), 23. Phenylpropanoid biosynthesis (ko00940), 24. Glycosylphosphatidylinositol (GPl) anchor biosynthesis (ko00563), 25. N-Glycan biosynthesis (ko00510), 26. Cysteine and methionine metabolism (ko00270), 27. ABC transporters (ko02010), 28. cGMP-PKG signaling pathway (ko04022), 29. Calcium signaling pathway (ko04020), 30. cAMP signaling pathway (ko04024), 31. MAPK signaling pathway (ko04010), 32. insulin resistance (ko04931), 33. Choline metabolism in cancer (ko05231), 34. Chemical carcinogenesis-receptor activation (ko05207), 35. Linoleic acid metabolism (ko00591), 36. Retinol metabolism (ko00830), 37. Starch and sucrose metabolism (ko00500), 38. Glycerophospholipid metabolism (ko00564), 39. Vitamin digestion and absorption (ko04977), 40. PPAR signaling pathway (ko03320), 41. Mineral absorption (ko04978), 42. Parathyroid hormone synthesis, secretion and action (ko04928), 43. Aldosterone synthesis and secretion (ko04925), 44. Bile secretion (ko04976), 45. Adrenergic signaling in cardiomyocytes (ko04261).

In total, 321 KEGG pathways were identified. The top 20 significantly enriched pathways are shown in Figure 7A, while the top 20 upregulated and downregulated pathways are presented in Figure 7B and Figure 7C, respectively. Specifically, 58 DEGs were mapped to Lysosome (ko04142), 55 to Protein processing in the endoplasmic reticulum (ko04141), 46 to Spliceosome (ko03040), 42 to Cell cycle (ko04110), 41 to Purine metabolism (ko00230), 31 to Nucleotide excision repair (ko03420), 29 to Amino sugar and nucleotide sugar metabolism (ko00520), 28 to Cell cycle–yeast (ko04111), 26 to DNA replication (ko03030), 24 to Base excision repair (ko03410), 22 to Glycine, serine and threonine metabolism (ko00260), 20 to Proteasome (ko03050), 20 to Tyrosine metabolism (ko00350), 18 to Alanine, aspartate and glutamate metabolism (ko00250), 16 to Mismatch repair (ko03430), 15 to Isoquinoline alkaloid biosynthesis (ko00950), 15 to Antigen processing and presentation (ko04612), 11 to Protein export (ko03060), 10 to Circadian rhythm (ko04710), and 9 to One carbon pool by folate (ko00670).

## 3. Discussion

Temperature is a key environmental factor regulating embryonic development in *S. paramamosain* [24,25]. In the present study, no significant differences were observed between maternal and in vitro hatching at the same temperature, indicating that in vitro hatching does not alter the intrinsic developmental rhythm of *S. paramamosain* embryos. However, both excessively low and high temperatures inhibited successful hatching into zoea larvae. These results are consistent with a previous report [25], who found that embryos hatched in vitro within the 20–30 °C range developed normally, whereas exposure to extreme temperatures of 10 °C or 35 °C resulted in abnormal cleavage and developmental arrest.

Embryonic development of *S. paramamosain* was clearly temperature-dependent, with embryos requiring 447 h to reach the ZI stage at 21 °C, but only 267 h at 33 °C. This strong temperature-development relationship underscores the critical role of temperature regulation in maintaining hatching efficiency. Developmental abnormalities and mortality increased markedly under extreme thermal conditions, consistent with previous findings that exposure to either high (35 °C) or low (10 °C) temperatures disrupts embryogenesis [25]. These observations suggest that maintaining hatching temperature within an optimal range is essential for ensuring efficient embryonic development and producing high-quality larvae [24].

Beyond developmental timing, comparisons between hatching modes have important practical implications. In vitro hatching provides a standardized and controllable platform that eliminates variability associated with maternal behavior, thereby facilitating high-throughput experimental designs for assessing environmental stress, investigating genetic and developmental mechanisms, performing toxicological evaluations, and supporting seedstock production. Although in vitro hatching does not accelerate embryogenesis, it offers reproducible conditions that enhance the reliability and comparability of the experimental results. Moreover, the findings indicate that in vitro hatching does not alter the developmental rate of *S. paramamosain* embryos. Differences between hatching modes could be attributed to the presence or absence of maternal assistance during hatching. Under maternal conditions, fanning of the abdominal appendages and movement of the pereiopods generate hydrodynamic forces that reduce the mechanical threshold for membrane rupture, thereby promoting synchronized hatching. In contrast, embryos hatched in vitro rely solely on endogenous mechanisms, particularly the localized hardening of dorsal and caudal spines, to actively perforate the chorionic membrane. This additional requirement for exoskeleton formation may slightly prolong the overall hatching period. Future studies integrating physiological and molecular approaches are warranted to further elucidate the mechanisms underlying developmental arrest at extreme temperatures and the biomechanical processes involved in membrane rupture under different hatching conditions.

In this study, temperature exerts a significant influence on the in vitro development of *S. paramamosain* embryos. With an optimal range of 27–30 °C, embryos successfully completed early cleavage and developed to the ZI, exhibiting markedly higher survival rates compared with other temperature treatments. These findings are consistent with observations in *P. trituberculatus*, in which embryos exhibited greater survival and hatching success under moderate temperatures, whereas extreme high or low temperatures substantially reduced viability [11]. Similarly, a thermal window of 27–30 °C has been reported to support optimal larval growth and survival in *S. paramamosain*, while embryogenesis slows considerably at 18–21 °C and typically arrests at the blastula stage [26]. Conversely, high temperatures (33–36 °C) accelerated the onset of eye pigmentation; however, survival rates declined sharply. Elevated temperatures have been shown to cause abnormal cell division and increase mortality by disrupting energy metabolism and cellular homeostasis [11,25]. Furthermore, adverse effects of high temperatures on mitochondrial function and ATP production have been documented in crustacean and other animal embryos [27], which may partly explain the rapid embryonic mortality observed under high-temperature treatments in the present study.

In the artificial seedling rearing of *S. paramamosain*, embryos from deceased ovigerous females are typically discarded. In vitro hatching provides a practical solution to several operational challenges, including low hatching rates, asynchronous larval emergence, and the loss of fertilized eggs due to maternal mortality [28]. Moreover, in vitro hatching allows for precise environmental control and mitigates losses associated with aggressive behavior, disease, or maternal death [29]. In the present study, when harvested within 4 h after maternal death, embryos achieved in vitro hatching success comparable to those collected from live females, suggesting no compromise in developmental potential. Consistent with previous observations on embryonic morphology and cell division dynamics, early- to mid-stage embryos exhibited sufficient structural robustness to continue normal development under controlled conditions, provided that they were collected promptly and maintained at appropriate temperatures [25,30]. Therefore, collecting embryos from recently deceased ovigerous females preserves embryos with high hatching potential, reduces broodstock losses, and improves overall efficiency in *S. paramamosain* seedstock production. Furthermore, the present findings demonstrate that embryos attached to the maternal pleopods of *S. paramamosain* are capable of independent hatching, supporting the absence of a direct maternal-embryo nutrient conduit or any direct maternal regulation of embryogenesis.

Significant enrichment was detected in the Cell cycle (ko04110, 42 DEGs) and Cell cycle-yeast (ko04111, 28 DEGs) pathways, reflecting regulatory processes associated with cell cycle progression and developmental control. Although these pathways are annotated with reference to animals and yeast, their core regulatory mechanisms are evolutionarily conserved and primarily mediated by the Cyclin-CDK axis, which orchestrates the temporal progression of events across the G1, S, G2, and M phases. Both pathway annotations and recent studies indicate that Cyclin-CDK complexes promote DNA replication during the S phase and mitosis during the M phase through stage-specific substrate phosphorylation, thereby ensuring orderly cell division and accurate chromosome segregation [31,32]. These results provide a molecular framework for the observed differences in cell cycle progression and proliferative activity between ZI larvae derived from in vitro and maternal hatching, which may account for variations in developmental duration and accumulated thermal units.

Crustaceans undergo extensive tissue remodeling and organogenesis during the pre-hatching and early post-hatching stages, including the formation of compound eyes, musculature, and exoskeletal structures, which are characterized by intensive cell proliferation and distinct transcriptomic activity. Consistent with these observations, studies on embryos and larvae of freshwater ornamental shrimp and the Chinese mitten crab have reported strong activation of genes involved in cell proliferation and protein synthesis during this critical developmental window [33]. During maternal hatching, ovigerous females provide external mechanical assistance through abdominal pleopod fanning and pereiopod movements, which likely reduce the mechanical threshold required for membrane rupture and promote synchronized larval emergence. In contrast, in vitro-hatched ZI lack maternal support and must rely on the prior formation and hardening of localized exoskeletal structures, such as dorsal and caudal spines, to actively breach the chorionic membrane. This “hardening-before-rupture” mechanism imposes a biomechanical constraint, thereby prolonging the time to membrane rupture and extending the overall hatching period. Morphological evidence supporting this mechanism has been reported in studies on *S. paramamosain* embryogenesis, which documented progressive changes in cell division, organ primordium formation, and embryonic membrane morphology [19]. Investigations of molting blue crabs (*Callinectes sapidus*) have demonstrated that the Young’s modulus and tensile strength of the exoskeleton vary markedly across different hardening stages, emphasizing the close relationship between exoskeletal hardening and mechanical resistance [34]. Furthermore, reviews on crustacean exoskeletal structural diversity indicate that the chitin-protein composite architecture and the degree of mineralization or hardening determine exoskeletal stiffness and shell strength, which likely influence the structural rigidity required for successful embryonic membrane rupture [35]. In crustaceans, exoskeleton formation has been linked to the coordinated reprogramming of amino acid and lipid metabolism. This includes the provision of amino sugars for chitin synthesis, amino acids for cuticular protein assembly, and catechol-mediated sclerotization. Activation of the hexosamine biosynthetic pathway (HBP) provides UDP-N-acetylglucosamine (UDP-GlcNAc), a key substrate for chitin synthase-mediated polymerization [36]. In parallel, amino acid metabolism supplies substrates for matrix proteins and supports antioxidant defense. Sclerotization is further dependent on phenoloxidase (PO) activity, which catalyzes the oxidation of DOPA and dopamine derivatives into quinones, promoting protein crosslinking that imparts pigmentation and exoskeletal rigidity [37,38]. In the present study, in vitro-hatched Z1 larvae exhibited a distinct “skeleton-first” metabolic profile, consistent with previous observations. The Amino sugar and nucleotide sugar metabolism pathway (ko00520) was significantly upregulated, suggesting enhanced flux through the hexosamine biosynthetic pathway to produce UDP-N-acetylglucosamine (UDP-GlcNAc) for chitin deposition. Concurrently, the Alanine, aspartate and glutamate metabolism (ko00250), Glycine, serine and threonine metabolism (ko00260), and Cysteine and methionine metabolism (ko00270) pathways were upregulated, providing amino acid substrates for matrix protein synthesis and one-carbon units for nucleotide biosynthesis and methylation. The cysteine/methionine-glutathione axis likely contributed to mitigating oxidative stress associated with catechol oxidation during cuticle hardening. In addition, Tyrosine metabolism (ko00350) was upregulated, reflecting increased demand for DOPA- and dopamine-derived catechols that facilitate quinone-protein crosslinking during exoskeletal sclerotization. Collectively, these results indicate that in vitro-hatched zoeae activate a metabolic program that prioritizes exoskeleton formation, integrating chitin biosynthesis, amino acid provisioning, and catechol-mediated sclerotization. This metabolic signature aligns with established mechanisms of crustacean cuticle assembly and demonstrates that in vitro hatching preserves the fundamental metabolic framework underlying exoskeleton development.

Conversely, Glycerophospholipid metabolism (ko00564) and Choline metabolism in cancer (ko05231), representing the choline-phospholipid axis, were relatively downregulated, suggesting that vesicular transport and membrane expansion were not prioritized at this developmental stage. Instead, metabolic resources appeared to be preferentially directed toward the amino sugar-chitin-matrix protein pathway. Although exogenous phospholipids are indispensable for growth, stress tolerance, and basal metabolism in crustacean larvae and juveniles, their demand and transcriptional activity are highly stage-specific—typically upregulated during stress responses or functionalization phases but transiently suppressed during the skeleton-first developmental window [39]. Similarly, Linoleic acid metabolism (ko00591), which contributes to the arachidonic acid-eicosanoid cascade and regulates membrane fluidity and signaling, was also downregulated, further reflecting stage-specific prioritization of cuticle assembly and sclerotization. In *S. paramamosain*, dietary arachidonic acid has been shown to promote cholesterol utilization, lipid deposition, and molting performance, suggesting that the lipid metabolic axis assumes greater importance during later functionalization and molting stages [40].

In the absence of maternal mechanical assistance during in vitro hatching, the observed upregulation of amino acid metabolism and concurrent downregulation of lipid-related pathways reflect a molecular strategy through which ZI larvae prioritize chitin monomer production, matrix protein synthesis, and phenoloxidase-mediated sclerotization prior to active membrane rupture. At this stage, membrane lipid remodeling and polyunsaturated fatty acid-mediated signaling are temporarily de-emphasized. This stage-specific reallocation of metabolic resources is consistent with recent findings describing the temporal sequence of shell formation, sclerotization, and lipid utilization during crustacean development [36,37,39,40,41].

Transcriptomic analysis revealed significant enrichment of multiple key pathways that collectively form a rapid-response cascade. MAPK signaling (ko04010) and Calcium signaling (ko04020) were strongly activated, underscoring their critical roles in rapid signal amplification and precise cellular modulation. In crustaceans, MAPK cascades downstream of pattern recognition receptor (PRR) activation, particularly via the MKK4/MKK6-p38 axis, regulate diverse immune outputs, whereas calcium signaling directly influences hemocyte vesicle trafficking and exosome secretion [42,43]. Enrichment of the Lysosome pathway (ko04142) suggests activation of phagosome-lysosome and/or autophagosome-lysosome degradation mechanisms, consistent with evidence that lysosome-dependent autophagy is a common stress or pathogen-induced response in crabs [44]. Similarly, enrichment of the Proteasome pathway (ko03050) highlights its role in protein quality control and the fine-tuning of immune signaling. Previous studies have shown that modulation of proteasomal activity can occur in a tissue-specific manner, either amplifying or attenuating innate immune responses [45]. Enrichment of the Protein export pathway (ko03060) indicates elevated activity along the endoplasmic reticulum-Golgi-vesicle secretion axis, likely reflecting enhanced exocytosis of antimicrobial peptides such as crustins and anti-lipopolysaccharide factors (ALFs). Recent studies have identified new members of the crustin family, underscoring their expanding functional repertoire in crustacean immunity [46]. In parallel, upregulation of ABC transporters (ko02010) suggests increased transmembrane efflux of pathogen-associated molecules or toxins, facilitating pathogen clearance and restoration of cellular homeostasis. This observation aligns with genomic and functional evidence from *Litopenaeus vannamei*, where multiple ABC transporters contribute to *Vibrio* resistance and modulate NF-κB signaling [47]. Collectively, these results indicate that in vitro-hatched ZI mount a robust innate immune program encompassing signal transduction (MAPK and Calcium signaling), pathogen clearance (Lysosome and Proteasome), and effector secretion (Protein export and ABC transporters). These findings highlight both the evolutionary conservation of rapid-response immune modules in crustaceans and the ability of embryos hatched outside the maternal body to activate immune responses comparable to those under maternal conditions. The enrichment of these immune-related pathways can likely be attributed to conditions encountered during in vitro hatching. Under artificial hatching conditions, embryos often settle unevenly and remain in prolonged contact with detritus at the bottom of the culture system, thereby increasing microbial exposure and triggering rapid innate immune activation. By contrast, maternal brooding behaviors, such as abdominal fanning and egg cleaning, generate localized water flow that removes debris and parasites, reducing direct egg-microbe interactions and resulting in lower levels of immune pathway activation. This “parental care” effect has been quantitatively demonstrated in lobsters, where its intensity increases throughout embryogenesis and contributes to synchronized hatching [48].

Furthermore, ABC transporters (ko02010) function as energy-dependent transmembrane “valves” that mediate solute exchange under the condition of salinity fluctuation, thereby cooperating with other ion channels to maintain hemolymph homeostasis [47]. Concurrently, the Calcium signaling pathway (ko04020) acts as a rapid “commander,” converting external stimuli into immediate adjustments in ion transport. This coordination enables precise regulation of osmoregulation over short timescales [43]. Embryos of *S. paramamosain* hatched in vitro may employ osmoregulatory strategies distinct from those operating under maternal brooding conditions. However, the precise molecular mechanisms and temporal coordination underlying these differences remain to be elucidated. Future studies should integrate functional assays of key transporters, including ABCB/ABCG, Na^+^/K^+^-ATPase, and V-type H^+^-ATPase, together with intracellular Ca^2+^ imaging and exosome-release analyses, to experimentally validate the regulatory pathways inferred from transcriptomic evidence.

## 4. Materials and Methods

### 4.1. Ethics Statement

All animal experiments in this study were conducted in accordance with the relevant national and international guidelines. Our project was approved by the East China Sea Fisheries Research Institute, Chinese Academy of Fishery Sciences (approval number 20250410-1). Our study did not involve endangered or protected species.

### 4.2. Experimental Animals and Sample Collection

Healthy broodstock (100 females, with an average weight of 350 ± 50 g and 8 months of age) were provided by the Innovation Team for Genetic Breeding of *S. paramamosain* at the Ninghai Aquatic Experimental Center, East China Sea Fisheries Research Institute, Chinese Academy of Fishery Sciences, Zhejiang Province, China. Broodstock were temporarily maintained in a concrete tank measuring 4 m in length, 8 m in width, and 0.6 m in water depth. The tank was shaded with netting and continuously aerated using air pumps. Water temperature was maintained at 26–28 °C and salinity at 25 ppt. Fresh razor clams were provided daily in the evening as feed, and approximately one-third of the water was renewed each day to maintain optimal water quality. After oviposition, ovigerous females were transferred to black cylindrical hatching barrels (80 cm in both height and diameter; volume approximately 400 L). The temperature and salinity in the barrels were kept consistent with those in the holding tank, and half of the water was replaced daily to remove uneaten feed and excreta.

Embryos were incubated in vitro using a custom-designed hatching apparatus. The system consisted of an upper cylindrical chamber and a lower inverted conical base, each measuring 50 cm in height and diameter, with a total volume of approximately 130 L. A circular air stone was positioned at the bottom to provide continuous aeration, ensuring embryo suspension and preventing mortality due to sedimentation or localized hypoxia. During hatching, direct sunlight was avoided, one-half of the water was replaced every other day, salinity was maintained at 25 ppt, and temperature was controlled according to the experimental design until the emergence of Zoea I (ZI) larvae. To compare the hatching time and hatching rate of ZI between in vitro and maternal hatching, five healthy ovigerous females carrying full egg masses were randomly selected. Specifically, embryos were detached using alcohol-sterilized forceps and collected at the newly fertilized pre-cleavage (1-cell) stage (before the first cleavage), which was confirmed by microscopic inspection. Approximately 1200 embryos were collected from each female after microscopic inspection. To ensure sterile conditions during sample collection, all instruments used (including forceps, scissors, and containers) were pretreated by immersion in 70% ethanol and subsequently autoclaved. Additionally, the embryos were carefully detached under sterile conditions using alcohol-sterilized forceps. All solutions were prepared with DNA/RNase-free water to maintain an enzyme-free environment. All embryos were transferred to the in vitro hatching apparatus and incubated at five experimental temperatures (21, 24, 27, 30, and 33 °C), with 18 °C and 36 °C included as supplementary extremes. After partial removal of eggs for in vitro hatching, the corresponding females, which still carried a substantial number of fertilized eggs on their abdominal appendages, were maintained in separate hatching buckets under identical temperature conditions for natural (maternal) hatching as controls. Salinity was consistently maintained at 25 ppt, and half of the water was replaced every 24 h. During the experimental period, samples were randomly collected at regular intervals, with a sampling interval of 2 h. At each sampling point, approximately ten embryos were observed. Morphological characteristics of embryos at the fertilized-egg, cleavage, blastula, gastrula, compound-eye pigmentation, non-segmented larval, and membrane-enclosed Zoea stages, following the staging criteria described by Xu et al. [19], were examined under a microscope and documented photographically. Additionally, embryo viability following maternal death was evaluated by selecting ovigerous females at different developmental stages. Egg masses were collected from deceased females at the blastula, non-segmented larval, compound-eye pigmentation, and near-hatching stages at 0, 2, 4, and 8 h after death. The collected embryos were immediately transferred to the in vitro hatching apparatus maintained at 27 °C and a salinity of 25 ppt. Aeration and water replacement were performed as described above. During hatching, samples were randomly collected for microscopic examination. Morphological changes were documented, developmental progression was recorded, and hatching success was calculated for each post-mortality sampling interval.

For transcriptomic analysis, larvae were collected from both the in vitro and maternal hatching groups. The in vitro group (IVI) consisted of ZI larvae derived from embryos incubated at 27 °C and a salinity of 25 ppt from the fertilized-egg stage, while the maternal group (MI) comprised larvae naturally hatched under the same environmental conditions. Three biological replicates were prepared for each group. Upon hatching, 600 larvae were randomly collected from each group and evenly divided into three replicates, with 200 individuals per replicate. The larvae were gently blotted with absorbent paper to remove surface water and immediately immersed in RNA preservation solution. Samples were stored at 4 °C overnight and subsequently transferred to −80 °C for long-term storage prior to transcriptome sequencing.

### 4.3. Data Analysis of Embryonic Development

Experimental data were presented as mean ± standard deviation (SD). Differences in hatching time and hatching rate among temperature treatments and hatching modes were analyzed using one-way analysis of variance (ANOVA) in SPSS version 23.0, followed by Tukey’s post hoc multiple comparison test. Graphs were generated using GraphPad Prism version 8.0. Line charts were used to visualize developmental duration and hatching rate trends across different temperature treatments.

### 4.4. Total RNA Extraction

Total RNA was extracted from six samples representing the two groups using TRIzol Reagent (Invitrogen, Waltham, MA, USA) following the manufacturer’s protocol. All instruments and materials, including scissors, forceps, steel beads, pipette tips, and centrifuge tubes, were pretreated by immersion in 0.1% diethyl pyrocarbonate (DEPC)-treated water and subsequently autoclaved to eliminate RNase contamination. All solutions were prepared with RNase-free water to maintain an RNase-free environment. RNA integrity was evaluated by 2% agarose gel electrophoresis. RNA purity was determined using a NanoDrop spectrophotometer (Thermo Fisher Scientific, Wilmington, DE, USA), and RNA concentration was measured with the Qubit RNA Assay Kit on a Qubit 2.0 Fluorometer (Life Technologies, Carlsbad, CA, USA). RNA quality was further verified using an Agilent 2100 Bioanalyzer (Agilent Technologies, Santa Clara, CA, USA).

### 4.5. cDNA Library Construction and Sequencing

Total RNA that passed all quality control criteria was used for cDNA library construction. For each sample, at least 1 μg of total RNA was processed using the Illumina NEBNext^®^ Ultra™ RNA Library Prep Kit (NEB, Ipswich, MA, USA). Library preparation included mRNA enrichment, fragmentation, double-stranded cDNA synthesis, PCR amplification, and quality assessment. The resulting libraries were sequenced by Shanghai Ouyi Biotechnology Co., Ltd. (Shanghai, China). Sequencing was performed using the Illumina NovaSeq 6000 platform (Illumina, San Diego, CA, USA) with paired-end reads of 150 bp.

### 4.6. Assembly and Functional Annotation

To ensure the reliability of downstream analyses, raw reads were processed using fastp v0.19.3 [49] to remove adapter sequences, low-quality bases, and reads containing excessive unknown nucleotides (N), thereby generating high-quality clean reads. The clean reads were subsequently assembled into transcripts with StringTie v2.1.2 [50], which employs a network flow algorithm and supports optional de novo assembly. Clean reads were aligned to the *S. paramamosain* reference genome (https://www.ncbi.nlm.nih.gov/datasets/genome/GCF_035594125.1/, accessed on 15 August 2025) using HISAT2 v2.2.1 [51] to obtain positional information and identify sequence features specific to each group. Novel gene sequences identified from the genome were functionally annotated using DIAMOND v0.9.24 [52] against the KEGG [53], GO [54], NR (NCBI, https://www.ncbi.nlm.nih.gov/refseq/, accessed on 17 August 2025), and Swiss-Prot (UniProt, https://www.uniprot.org/, accessed on 17 August 2025) databases to provide comprehensive functional characterization.

### 4.7. Gene Expression Quantification

Gene identification was based on alignment coverage of at least 80% and a minimum identity percentage of 95%. The gene alignment results were quantified using featureCounts v1.6.2 [55], and gene expression levels were calculated as fragments per kilobase of transcript per million mapped reads (FPKM). This normalization metric accounts for both sequencing depth and gene length, providing a reliable measure of transcript abundance. Principal component analysis (PCA) was conducted across all samples to reduce data dimensionality while preserving the primary sources of variance, thereby facilitating the interpretation of overall gene expression patterns.

### 4.8. Differential Expression Analysis

Clean reads were aligned to the *S. paramamosain* reference genome using HISAT2. Differential gene expression between the IVI and MI groups was analyzed using DESeq2 v1.22.1, and *p*-values were adjusted using the Benjamini-Hochberg false discovery rate (FDR) correction. Genes with a fold change greater than 1 and an adjusted *p*-value < 0.05 were identified as differentially expressed genes (DEGs). Volcano plots were generated to visualize the overall distribution of DEGs. In addition, hierarchical clustering analysis was performed to examine expression patterns across groups, with genes exhibiting similar expression profiles grouped together to infer potential functional relationships and novel roles of uncharacterized genes.

### 4.9. Functional Enrichment Analysis of DEGs

Following the identification of differentially expressed genes (DEGs), Gene Ontology (GO) enrichment analysis was performed to quantify the number of DEGs associated with each GO term and to generate the corresponding gene lists. This analysis enabled the identification of GO terms significantly enriched with DEGs, thereby revealing the major biological functions represented. In addition, Kyoto Encyclopedia of Genes and Genomes (KEGG) pathway enrichment analysis was conducted using a hypergeometric test. The KEGG database serves as an integrated platform linking genomic information with higher-order functional pathways, allowing for comprehensive exploration of gene interactions and expression networks.

## 5. Conclusions

This study systematically examined the developmental characteristics of *S. paramamosain*. embryos under varying temperatures, hatching modes, and maternal mortality conditions, integrating transcriptomic analyses to elucidate the underlying functional mechanisms. The results demonstrated that embryos exhibited moderate developmental rates and the highest survival at 27–30 °C, identifying this range as optimal for successful hatching. Low temperatures (≤21 °C) delayed embryogenesis, whereas high temperatures (≥33 °C) markedly reduced survival and were frequently accompanied by pathogen proliferation. Both in vitro and maternal hatching followed largely comparable developmental trajectories under suitable environmental conditions. It was found that embryos collected within the 4 h window after maternal death were capable of successful hatching, thereby supporting the use of in vitro systems to utilize maternal resource. Collectively, these results indicate the absence of a direct maternal-embryo nutrient conduit and direct maternal regulation of embryogenesis in *S. paramamosain*. Transcriptomic analysis revealed that DEGs were primarily enriched in pathways related to cell cycle regulation and developmental control, energy metabolism and ion transport, immune responses, and osmoregulation. These findings highlight the molecular strategies by which embryos maintain genomic stability, secure energy supply, and preserve homeostasis under in vitro hatching conditions.

## Figures and Tables

**Table 1:** Total accumulated temperature (ATU) from embryo to Zoea I of *S. paramamosain* under in vitro and maternal conditions at different hatching temperatures.

Temperature (°C)	Hatching Mode	Accumulated Temperature (°C·h)
21	IVI	9387
	MI	9261
24	IVI	9120
	MI	9168
27	IVI	8883
	MI	8748
30	IVI	8730
	MI	8580
33	IVI	8811
	MI	8778

Note: IVI, in vitro hatching; MI, maternal hatching.

**Table 2:** Quality control (QC) of transcriptome sequencing data.

Sample	Raw Reads (M)	Raw Bases (G)	Clean Reads (M)	Clean Bases (G)	Valid Bases (%)	Q30 (%)	GC (%)
IVI1	49.91	7.32	48.15	7.06	96.48	96.25	38.89
IVI2	49.08	7.22	47.47	6.98	96.73	96.74	39.02
IVI3	48.87	7.13	46.75	6.82	95.67	96.36	47.73
MI1	48.79	7.15	46.96	6.88	96.26	96.43	47.98
MI2	41.98	6.12	40.07	5.84	95.44	96.03	49.62
MI3	42.93	6.28	41.25	6.04	96.09	96.41	50.33

Note: IVI, in vitro hatching; MI, maternal hatching.

## Data Availability

The datasets for this study can be found in the NGDC SRA Sequence Database at https://ngdc.cncb.ac.cn/gsub/submit/gsa/subCRA011652/overview, under reference number No. CRA010016, accessed on 11 August 2025.

## Supplementary Materials

The following supporting information can be downloaded at: https://www.mdpi.com/article/10.3390/ijms27020714/s1.

## References

[1] Bondarenko V., Nikolaev M., Kromm D., Belousov R., Wolny A., Blotenburg M., Zeller P., Rezakhani S., Hugger J., Uhlmann V. (2023). Embryo-uterine interaction coordinates mouse embryogenesis during implantation. EMBO J..

[2] Winiarczyk D., Khodadadi H., Leszczynski P., Taniguchi H. (2025). A simple validation and screening method for CRISPR/Cas9-mediated gene editing in mouse embryos to facilitate genetically modified mice production. PLoS ONE.

[3] Zhang Y., Yin T., Zhou L. (2023). CRISPR/Cas9 technology: Applications in oocytes and early embryos. J. Transl. Med..

[4] Jeong T.Y., Yoon D.E., Kim S.P., Yang J., Lim S.-Y., Ok S., Ju S., Park J., Lee S.B., Park S.-J. (2025). An innovative approach using CRISPR-ribonucleoprotein packaged in virus-like particles to generate genetically engineered mouse models. Nat. Commun..

[5] Hildebrandt T.B., Holtze S., Colleoni S., Hermes R., Stejskal J., Lekolool I., Ndeereh D., Omondi P., Kariuki L., Mijele D. (2023). In vitro fertilization program in white rhinoceros. Reproduction.

[6] Roth T.L. (2024). That was then, this is now–over two decades of progress in rhinoceros reproductive science and technology. Theriogenol. Wild.

[7] Perez-Atehortua M., Short S.E., Aranzaez-Rios C., Farias J., Oliveira R.P.S., Pereira W.A., Risopatron J., Valdebenito I., Villalobos E.F. (2024). Preparation and extraction of chorion proteins from Salmo salarembryos at the pigmented eye stage for electrophoresis with SDS-polyacrylamide gel. MethodsX.

[8] Piamsomboon P., Mehl N.S., Sirivaidyapong S., Wongtavatchai J. (2019). Assisted reproduction in Nile tilapia *Oreochromis niloticus*: Milt preservation, spawning induction and artificial fertilization. Aquaculture.

[9] Rbbani G., Siriyappagouder P., Murshed R., Joshi R., Nedoluzhko A., Galindo-Villegas J., Fernandes J.M.O. (2025). Optimization of Nile tilapia artificial breeding Using Human Chorionic Gonadotropin (hCG) Hormone. Methods Protoc..

[10] Xu C., Tabebordbar M., Iovino S., Ciarlo C., Liu J., Castiglioni A., Price E., Liu M., Barton E.R., Kahn C.R. (2013). A zebrafish embryo culture system defines factors that promote vertebrate myogenesis across species. Cell.

[11] He J., Wan L., Yu H., Peng Y., Zhang D., Xu W. (2022). Effect of water temperature on embryonic development of *Protunus trituberculatus* in an off-season breeding mode. Front. Mar. Sci..

[12] dos Santos J.R., Zadinelo I.V., Mauerwerk M.T., Ronnau M., Ballester E.L.C. (2024). Histological analysis of *Macrobrachium rosenbergii* (de Man) eggs incubated in different salinities. Lat. Am. J. Aquat. Res..

[13] Ma K., Liu Z., Qiao G., Ma L., Zhang F., Zhao M., Ma C., Wang W. (2023). Effects of four diets on the metabolism of megalopa metamorphosis of the mud crab, *Scylla paramamosain*. Front. Mar. Sci..

[14] Qiao N., Liu Z.Q., Li Y.Y., Zhang F.Y., Ma C.Y., Wang X.Y., Xu J.Y., Ma L.B., Ma K.Y., Wang W. (2025). An integrated transcriptomic and proteomic approach uncovers the molecular mechanisms of hypoosmotic adaptation in *Scylla paramamosain* megalopa. Int. J. Mol. Sci..

[15] Sui L., Wille M., Cheng Y., Wu X., Sorgeloos P. (2011). Larviculture techniques of Chinese mitten crab *Eriocheir sinensis*. Aquaculture.

[16] Wu X., Cheng Y., Zeng C., Wang C., Yang X. (2010). Reproductive performance and offspring quality of wild-caught and pond-reared swimming crab *Portunus trituberculatus* broodstock. Aquaculture.

[17] Lu Z., Shi C., Liu L., Mu C., Ye Y., Wang C. (2022). Phospholipid Compositions in *Portunus trituberculatus* Larvae at Different Developmental Stages. J. Ocean Univ. China.

[18] Wang W., Liu Z., Wang X., Zhang F., Ma C., Zhao M., Ma K., Ma L. (2024). Feeding rhythm of the zoea larvae of *Scylla paramamosain*: The dynamic feeding rhythm is not completely synchronized with photoperiod. Heliyon.

[19] Xu L., Ma K., Zhang F., Wang W., Ma L., Jin Z., Zhao M., Chen W., Fu Y., Ma C. (2023). Observations on the embryonic development of the mud crab, *Scylla paramamosain*. Front. Mar. Sci..

[20] Liew K.S., Yong F.K.B., Lim L.S. (2023). An overview of the major constraints in *Scylla* mud crabs grow-out culture and its mitigation methods. Aquac. Stud..

[21] Kasan N.A., Yee C.S., Manan H., Ideris A.R.A., Kamaruzzan A.S., Waiho K., Lam S.S., Mahari W.A.W., Ikhwanuddin M., Suratman S. (2021). Study on the implementation of different biofloc sedimentable solids in improving the water quality and survival rate of mud crab, *Scylla paramamosain* larvae culture. Aquac. Res..

[22] Nghia T.T., Wille M., Vandendriessche S., TheVinh Q., Sorgeloos P. (2007). Influence of highly unsaturated fatty acids in live food on larviculture of mud crab *Scylla paramamosain* (Estampador 1949). Aquac. Res..

[23] Kwok C.T.-K., Yu R.C.-W., Hau P.-T., Cheung K.Y.-C., Ng I.C.-F., Fung J., Wong I.T.-F., Yau M.C.-Y., Liu W.-M., Kong H.-K. (2024). Characteristics and pathogenicity of *Vibrio alginolyticus* SWS causing high mortality in mud crab (*Scylla serrata*) aquaculture in Hong Kong. Front. Cell. Infect. Microbiol..

[24] Xu L., Ma C., Zhang F., Wang W., Zhao M., Jin X., Yin J., Ma L., Chen W., Xu J. (2024). Embryonic genome activation (EGA) occurred at 1-cell stage of embryonic development in the mud crab, *Scylla paramamosain*, revealed by RNA-Seq. Mar. Biotechnol..

[25] Zeng C. (2007). Induced out-of-season spawning of the mud crab, *Scylla paramamosain* (Estampador) and effects of temperature on embryo development. Aquac. Res..

[26] Nguyen T.P., Nguyen T.E., Nguyen T.K.H., Le Q.V., Do T.T.H. (2021). Effects of different temperatures on the growth and survival of mud crab *Scylla paramamosain* larvae. CTU J. Innov. Sustain. Dev..

[27] Roth Z., Hansen P.J. (2004). Involvement of apoptosis in disruption of developmental competence of bovine oocytes by heat shock during maturation. Biol. Reprod..

[28] Syafaat M.N., Azra M.N., Waiho K., Fazhan H., Abol-Munafi A.B., Ishak S.D., Syahnon M., Ghazali A., Ma H., Ikhwanuddin M. (2021). A review of the nursery culture of mud crabs, genus *Scylla*: Current progress and future directions. Animals.

[29] Carral J.M., Sáez-Royuela M., Celada J.D., Pérez J.R., Melendre P.M., Aguilera A. (2003). Advantages of artificial reproduction techniques for white-clawed crayfish (*Austropotamobius pallipes* Lereboullet). Bull. Fr. Peche Piscic..

[30] Hamasaki K. (2003). Effects of temperature on the egg incubation period, survival and developmental period of larvae of the mud crab *Scylla serrata* (Forskal) (Brachyura: Portunidae) reared in the laboratory. Aquaculture.

[31] Basu S., Greenwood J., Jones A.W., Nurse P. (2022). Core control principles of the eukaryotic cell cycle. Nature.

[32] Pluta A.J., Studniarek C., Murphy S., Norbury C.J. (2024). Cyclin-dependent kinases: Masters of the eukaryotic universe. Wiley Interdiscip. Rev.-RNA.

[33] Yan C., Wu Z., Liu Y., Sun Y., Zhang J. (2024). Comparative transcriptomic analysis primarily explores the molecular mechanism of compound eye formation in *Neocaridina denticulata sinensis*. BMC Genom..

[34] Taylor J.R.A., Hebrank J., Kier W.M. (2007). Mechanical properties of the rigid and hydrostatic skeletons of molting blue crabs, *Callinectes sapidus* Rathbun. J. Exp. Biol..

[35] Vittori M. (2024). Structural diversity of crustacean exoskeletons and its implications for biomimetics. Interface Focus.

[36] Zhang X., Yuan J., Li F., Xiang J. (2021). Chitin synthesis and degradation in crustaceans: A genomic view and application. Mar. Drugs.

[37] Campli G., Volovych O., Kim K., Veldsman W.P., Drage H.B., Sheizaf I., Lynch S., Chipman A.D., Daley A.C., Robinson-Rechavi M. (2024). The moulting arthropod: A complete genetic toolkit review. Biol. Rev..

[38] Sugumaran M., Sugumaran M. (2022). Chapter two-cuticular sclerotization in insects-a critical review. Advances in Insect Physiology.

[39] Liu T., Xu H., Feng W., He J., Han T., Wang J., Wu Q., Wang C. (2024). Balancing act: How cholesterol and phospholipids influence juvenile mud crab *Scylla paramamosain* growth and lipid metabolism. Aquac. Rep..

[40] Xie S., Deng Y., Tang Z., Tian Y., Cao H., Zhan W., Zhu T., Shen Y., Zhao W., Peng H. (2024). Dietary arachidonic acid supplementation promoted cholesterol utilization, lipid deposition and molting for *Scylla paramamosain*. Aquaculture.

[41] Barua H., Acharjee M.R., Giteru S.G., Chowdhury M., Wu H., Kumar L., Ahmmed M.K. (2025). Dietary phospholipids and their Impact on crustacean physiology: Growth, metabolism, immunity, and beyond. Aquac. Nutr..

[42] Betancourt J.L., Rodriguez-Ramos T., Dixon B. (2024). Pattern recognition receptors in crustacea: Immunological roles under environmental stress. Front. Immunol..

[43] Lei Y., Zhang M., Huang K., Sun Q., Li J., Liang H., Zheng Y., Tran N.T., Chen X., Zhang Y. (2025). Ca^2+^-SpVAMP2 pathway promotes exosome secretion to resist the infection of *Vibrio parahaemolyticus* in mud crab (*Scylla paramamosain*). Aquaculture.

[44] Lu Y., Liu Y., Cao J., Zhang Y., Zheng Y., Wang F. (2025). Waterborne ammonia toxicity damages crustacean hemocytes via lysosome-dependent autophagy: A case study of swimming crabs *Portunus trituberculatus*. Environ. Res..

[45] Grover M., Gang S.S., Troemel E.R., Barkoulas M. (2024). Proteasome inhibition triggers tissue-specific immune responses against different pathogens in *C. elegans*. PLoS Biol..

[46] Zhang W., Wei L., Chen P., Ning B., Wang J., He P., Shang C., Yu D. (2024). Discovery and characterization of an atypical crustin antimicrobial peptide from *Pollicipes pollicipes*. Mar. Drugs.

[47] Luo S.-S., Chen X.-L., Wang A.-J., Liu Q.-Y., Peng M., Yang C.-L., Yin C.-C., Zhu W.-L., Zeng D.-G., Zhang B. (2024). Genome-wide analysis of ATP-binding cassette (ABC) transporter in *Penaeus vannamei* and identification of two ABC genes involved in immune defense against *Vibrio parahaemolyticus* by affecting NF-κB signaling pathway. Int. J. Biol. Macromol..

[48] Sisti A.R., Jellison B., Shields J.D., Rivest E.B. (2024). Brood-grooming behavior of American lobsters *Homarus americanus* in conditions of ocean warming and acidification. Mar. Ecol. Prog. Ser..

[49] Chen S., Zhou Y., Chen Y., Gu J. (2018). fastp: An ultra-fast all-in-one FASTQ preprocessor. Bioinformatics.

[50] Pertea M., Pertea G.M., Antonescu C.M., Chang T.-C., Mendell J.T., Salzberg S.L. (2015). StringTie enables improved reconstruction of a transcriptome from RNA-seq reads. Nat. Biotechnol..

[51] Kim D., Langmead B., Salzberg S.L. (2015). HISAT: A fast spliced aligner with low memory requirements. Nat. Methods.

[52] Buchfink B., Xie C., Huson D.H. (2015). Fast and sensitive protein alignment using DIAMOND. Nat. Methods.

[53] Kanehisa M., Sato Y., Kawashima M., Furumichi M., Tanabe M. (2016). KEGG as a reference resource for gene and protein annotation. Nucleic Acids Res..

[54] Ashburner M., Ball C.A., Blake J.A., Botstein D., Butler H., Cherry J.M., Davis A.P., Dolinski K., Dwight S.S., Eppig J.T. (2000). Gene Ontology: Tool for the unification of biology. Nat. Genet..

[55] Liao Y., Smyth G.K., Shi W. (2014). featureCounts: An efficient general purpose program for assigning sequence reads to genomic features. Bioinformatics.

